# Author index

**DOI:** 10.1054/bjoc.2001.1914

**Published:** 2001-06


					Aaltonen LA 1314
Abbruzzese A 1586
Abe N 1317
Abe T 824
Ablett S 11
Achard JL 31 (Suppl 2)
Achtelik W 1036
Adenis A 65 (Suppl 2)
Adolfsson J 329, 1505
Adrian TE 926
Agus V 1087
Ahn BW 844
Aiken R 157
Aird R 600
Akerley W 157
Akeshima R 1551
Akhmedkhanov A 975
Akiba H 920
Akre K 965
Ala-aho R 659
Albers P 1330
Albery IP 1172
Alcock HE 499
Alfaro M 1219
Allemann J 33
Allen J 157
Allinen M 116
Allredl DC 493
Alsner J 1070
Altermatt HJ 1372
Alvarnas J 1591
Amos CI 1182
Andersen A 397
Andersson SW 1193
Andre F 903
Andreoli F 878
Andrulis IL 1635
Angus B 270
Aoki M 1610
Appierto V 1528
Arab MO 226
Arakawa S 859
Arbuck S 1424
Ardavanis A 1488
Arditti J 604
Argenio GF 636
Armstrong A 1433
Arnogiannaki N 1488
Arnold JM 352
Aromaa A 122
Arora A 340
Arseneau J 17 (Suppl 1)
Asami H 1242
Aschele C 1023
Askling J 113
Asselain B 24 (Suppl 2)
Assouline D 1179
Atkinson R 170
Atomi Y 1317
Aubele M 202
Austrup F 1664
Autillo-Touati A 360
Azzarelli A 1087
Baccino FM 946
Bachelot T 18 (Suppl 2)
Baelde JJ 535
Bah E 1207
Bahl A 1143
Baidas S 157
Bailly C 31 (Suppl 2),
81 (Suppl 2)
Baldelli AM 470
Balducci A 722
Ballinger JR 367
Balmaceda I 1219
Banerjee S 975
Bar Meir S 475
Baria K 892
Barletta E 38
Barni S 470
Baron JA 714
Bartels T 813
Bartholomew C 1630
Barzelloni ML 38
Bassi C 253
Bassilopoulos P 1488
Basuyau JP 18 (Suppl 2), 24
(Suppl 2), 31 (Suppl 2)
Bataillard A 8 (Suppl 2), 24
(Suppl 2), 31 (Suppl 2)
Batchelor T 157
Batlle G 1215
Batsis C 1602
Bauchinger M 202
Baudin E 808
Bayer P 344
Bayoumy S 1182
Bearzatto A 1387
Beckendorf V 81 (Suppl 2)
Beckman RA 1043
Begent RHJ 1671
Beijnen JH 42
Belizario JE 1135
Bell CMJ 435
Bell RS 1635
Beloueche M 691
Belyakov OV 674
Ben Ayed F 783
Bender DP 1624
Bengtsson C 1193
Benhamiche AM 1477
Benharkat S 783
Benicke M 982
Bennouna J 1179
Bensadoun RJ 42 (Suppl 2)
Bentzen SM 1084
Bergenbrant S 621
Berger J 819
Bergeron C 360
Berggren P 1505
Berghmans T 1150
Berney DM 340
Bernhard J 1156
Bernini GP 636
Bertani H 881
Berthois Y 936
Bertler SZ 157
Bessell EM 1293
Best N 1482
Betticher DC 1372
Bevan S 1314
Bey P 55 (Suppl 2)
Bezold G 72
Bharaj B 760
Bhargava P 1599
Bianchi P 196
Bibby MC 290
Bible KC 1391
Bicknell D 1314
Bicknell R 558
Bidart JM 808
Bijker N 539
Bijman MNA 1115
Bilancia D 38
Birch JM 141
Birembaut P 1616
Bissett D 308
Bjorkholm M 621
Blackhall FH 465
Blanc-Vincent, MP 8 (Suppl 2),
37 (Suppl 2), 42 (Suppl 2)
Blay JY 78 (Suppl 2)
Blizzard L 392
Blomqvist C 704, 1047
Blomqvist CP 244
Boccia R 17 (Suppl 1)
Bocko D 1339
Boddy AV 11, 452, 1029
Bode U 1453
Bodmer WF 1314
Boehm DA 571
Boerner SA 1391
Boffi B 878
Boggi U 1535
Boher J-M 903
Boige V 631
Bokemeyer C 313
Bond JA 520
Bondy ML 1182
Bonnier P 783
Borner MM 1372
Borrello S 529
Bory J-P 1616
Bosch CAJ 539
Boshoff C 1008 (BR)
Boussin FD 631
Boven E 550
Bowman A 600
Bowman KJ 106
Boyle P 1472
Brada M 148 (BR)
Braithwaite KL 270
Bramald S 1002
Brampton M 11
Branle F 1150
Bratti MC 1219
Bremond A 24 (Suppl 2),
31 (Suppl 2)
Brindle KM 832
Brinkmann K 697
British National Lymphoma
Investigation and Central
Lymphoma Group 1293
Brosman S 24 (Suppl 1)
Browman G 1 (Suppl 2)
Brown JM 19
Brown NJ 1384
Browne JK 3 (Suppl 1)
Brugere J 37 (Suppl 2)
Brunat-Mentigny M 78
(Suppl 2)
Brunetti C 1166
Bubenik J 374
Bucana CD 844
Buckley TJD 1443
Buisson M 783
Bukowski R 24 (Suppl 1)
Bull JH 1512
Bull S 1635
Bullerdick J 381
Bunch K 1460
Burk RD 1219
Burniat A 1150
Burton A 1293
Buttafoco P 881
Buyse IM 743
Cabal-Manzano R 1599
Cacciatore B 897
Caldas C 193
Calogero A 1115
Calvert AH 106, 452
Calvert H 1443
Cameron DA 1146
Campbell NC 910
Campbell T 157
Cancer Research Campaign
Phase I/II Committee 465
Canevari S 1528
Cano J-P 1677
Capelli P 253
Capkun V 1227
Caputo S 1535
Caraglia M 1586
Carder PJ 1095
Author index to Volume 84
Key to abbreviations:
(BR) Book reviews; (L) Letters to the editors; (Suppl 1) Supplement 1; (Suppl 2) Supplement 2
1687
British Journal of Cancer (2001) 84(12), 1687-1694
(c) 2001 Cancer Research Campaign
doi: 10.1054/ bjoc.2001.1914, available online at http://www.idealibrary.com on
Carmichael J 1443
Caroti C 1023
Carreca I 1166
Carella AM 55 (Suppl 2)
Carrie C 78 (Suppl 2)
Cartwright RA 141
Casaretti R 470
Casciari JJ 1544
Cascinu S 470
Cassidy J 308, 910
Castaigne D 24 (Suppl 2)
Castle PE 1219
Catalano G 470
Catalano V 470
Catalin J 604
Caux V 482
Cavadini E 1528
Cavenagh JD 147 (L)
Cemazar M 565
Chamberlain MC 157
Chan D 24 (Suppl 1)
Chan FKL 335
Chaplin DJ 565, 626
Chastagner P 604
Chatelain P 360
Chatenoud L 722
Chauvel P 42 (Suppl 2)
Chauvergne J 24 (Suppl 2)
Chauvot P 78 (Suppl 2)
Chay KO 844
Chen H-L 1556
Chen J 1265
Chen Q 1520
Chen WY 441 (L)
Chen-Shtoyerman R 475
Chenevix-Trench G 352
Cheng KK 1215
Cherny N 587
Chilvers CED 413
Chin S-F 193
Chiu S-M 1099
Cho DY 186
Cho M-S 1252
Cho YK 186
Choi J-H 186
Choong PFM 951
Chow W 1591
Christen RD 1571
Christensen M 1084
Christian M 1424
Chuang LY 709
Chung SCS 335
Ciccarelli A 1586
Ciccolini J 1677
Cini G 878
Cioffi R 38
Cirillo M 1023
Ciszak L 1339
Clarke K 1322
Clarke NW 1417
Clavel C 1616
Clendeninn N 11
Cleris L 1528
Cleton-Jansen AM 535
Cluck MW 926
Coates AS 1156
Coebergh JWW 700
Cohen-Solal C 18 (Suppl 2)
Cohn A 157
Colangelo D 1387
Colella G 1387
Coleman R 170
Coleman RE 1126
Collard TJ 520
Collier M 308
Collingridge DR 626
Collins A 429
Collins HM 1664
Colowick AB 17 (Suppl 1),
24 (Suppl 1)
Colton T 126
Comella G 470, 1166
Comella P 1166
Commo F 504
Comoy E 808
Conroy T 65 (Suppl 2)
Corbo M 1397
Corcoran JJ 489
Cornbleet MC 1146
Corpet DE 90
Cortesi E 1023
Coshic O 1016
Cosset JM 55 (Suppl 2)
Costa A 651
Costelli P 946
Cotter FE 1397
Cowan R 8
Craft JA 1630
Cramer DW 714
Cranston D 558
Crawford M 170
Creaney J 52
Crepin M 802
Croucher PI 1126
Cruickshank M 170
Cull A 594
Cullen MH 1293
Cummings J 600
Cummins C 1215
Cunningham D 1
Cupissol D 81 (Suppl 2)
Cuq P 1677
Curtin NJ 106
Cutuli B 55 (Suppl 2)
Dabit D 504
Dachez R 360
Dai Q 994
Daidone MG 651, 1387
Dalley CD 147 (L)
Dame MK 1076
Dancourt V 1477
Danesi R 1535
d'Anjou J 31 (Suppl 2)
Darby S 728
Dassonville O 42 (Suppl 2)
Dauplat J 18 (Suppl 2)
Davies MPA 1064
Day N 423
de Conno F 587
de Haes JCJM 1577
De Jaeger K 1280
de Jong S 1348
de Jonge MWA 1115
de Klerk N 52
de Leij L 1115
de Leij LFMH 1348
De Lena M 651
De Paoli P 122
De Ridder M 1122
De Tullio R 946
de Vries EGE 1348
Debuiche S 8 (Suppl 2)
DeCataldis G 1166
Decker S 1424
Deguchi H 915
Del Tacca M 1535
Delahaye L 1115
Delattre O 199
Dellapasqua S 651
Demard F 37 (Suppl 2),
42 (Suppl 2)
Demetri G 17 (Suppl 1)
Demetri GD 31 (Suppl 1)
Depierre A 903
D'Erasmo E 636
Dermime S 1433
Descamps P 31 (Suppl 2)
DeVries A 179
Dey P 8
Di Paolo A 1535
Di Pietro A 1405
Diamandis EP 643, 760
Dick GS 1029
Diehl V 381
Diel IJ 344
Dillner J 122
Diociaiuti L 460
Dodd HE 141
Doll R 728
Dombernowsky P 1301
Dong C 388
Dore JF 81 (Suppl 2)
Doroshow JH 1591
Dorval T 81 (Suppl 2)
dos-Santos-Silva I 406
Douillard JY 1179
Doz F 604
Drummond F 218
Dubois JB 24 (Suppl 2)
Dubreuil A 579
Duchateau L 539
Duffy S 423
Dugani A 881
Dummer R 1036
Durand-Zaleski I 4
(Suppl 2)
Durrant LG 80, 1443
Duval C 539
Duvillard P 18 (Suppl 2)
Dwivedi SN 1016
Dwyer T 392
Eardley A 8
Earle CC 441 (L)
East Anglian Breast Screening
Programme 423
Easton D 388
Edwards YH 218
Eerola H 116, 704
Eggleton P 170
Egrie JC 3 (Suppl 1)
Ehrhardt A 813
Eisenbrand G 283
Eissa S 1182
Ekbom A 113
Ekstrom AM 965
El-Abbadi M 1107
El-Badawy SA 1182
Elgie AW 680
El-Hariry I 1656
Elias D 74 (Suppl 2)
Elliott AM 910
Elliott P 1482
Ellis LM 844
Ellison G 1512
Ellwanger U 1036
Elomaa I 1047
Eng C 748
Engert D 202
Esteve J 1477
Estlin EJ 11, 1029
Etienne M-C 579
European Lung Cancer Working
Party (ELCWP) 1150
Evans HS 435
Evrard A 1677
Exton MS 1265
Fait E 1354
Faivre J 1477
Faivre S 631
Falconi M 253
Falk S 1143
Fallowfield L 48, 1011
Fang W 743
Farsi F 8 (Suppl 2),
31 (Suppl 2), 78 (Suppl 2)
Faucher A 37 (Suppl 2)
Fels LM 313
Fennell DA 1397
Fenwick J 452
Ferdeghini M 636
Feretti I 881
Ferme C 55 (Suppl 2)
Fernandez E 722
Fervers B 8 (Suppl 2),
18 (Suppl 2), 24 (Suppl 2),
31 (Suppl 2), 51 (Suppl 2),
55 (Suppl 2), 78 (Suppl 2),
81 (Suppl 2)
Fey M 1036
Fidder HH 475
Fielding K 1443
Figer A 475, 478
Fina F 783
Fischel J-L 579
Fisher JL 951
Flavell DJ 571
Flavell SU 571
Fleischhack G 1453
Fleishman A 24 (Suppl 1)
Fleming D 170
Flex D 478
Foekens JA 783
Folkard M 674
Fonatsch C 381
Fonderico F 1586
Fondrinier E 24 (Suppl 2),
31 (Suppl 2)
Foray N 1272
Forman SJ 1591
Formelli F 1528
Formento P 579
Fouret P 504
Fracchioli S 643
Franceschi S 722
Francesconi D 878
Francis RJ 1671
Francois E 61 (Suppl 2)
1688 Author index
British Journal of Cancer (2001) 84(12), 1687-1694 (c) 2001 Cancer Research Campaign
Frappaz D 604
Frasci G 1166
Frassineti GL 1023
Friedel A 283
Friedman E 475, 478
Frontini L 38, 470
Fry A 594
Frydecka I 1339
Fuchimoto S 443
Fueyo J 1252
Fuggle S 558
Fujita H 836
Fujita T 1564
Fujita Y 824, 836
Fukiyama C 558
Fukuda R 1640
Fulton D 157
Fulton R 1630
Gabriel R 1616
Gainet M 903
Galeotti T 529
Gallateau-Salle F 49 (Suppl 2)
Gallick GE 844
Gallo C 38
Gallo L 1023
Gao F 1185
Gao Y-T 994
Garbay JR 81 (Suppl 2)
Garbe C 1036
Gaumann A 1354
Gauthier LR 631
Gayko U 24 (Suppl 1)
Gebski V 297
Geick A 813
Gelleret N 87
Gennari L 196
Gentet JC 78 (Suppl 2), 604
Genzlinger A 283
George NJR 1417
Gerard H 783
Gercel-Taylor C 1624
Gerhold M 1571
Gerken M 134
Geva R 478
Ghisdal L 1150
Giai M 760
Giammarile F 55 (Suppl 2),
78 (Suppl 2)
Guillo S 8 (Suppl 2)
Giannini F 881
Giannoudis A 1058
Gibbs A 8
Gilbert M 157
Gill L 1460
Giovannini M 74 (Suppl 2)
Girling DJ 1447
Giusti C 936
Glantz MJ 157
Glaspy J 1 (Suppl 1),
17 (Suppl 1), 24 (Suppl 1)
Glavina-Durdov M 1227
Gochi A 443
Goepel JR 499
Gokgoz N 1635
Goldacre MJ 1460
Gomez-Manzano C 1252
Gordini G 643
Gordon D 17 (Suppl 1)
Gore M 1043, 1146
Gory G 8 (Suppl 2)
Gory-Delabaere G 37 (Suppl 2),
42 (Suppl 2), 51 (Suppl 2)
Goto A 1317
Graadt van Roggen JF 535
Graham J 170
Granotier C 631
Grau B 1179
Grazia Caprio M 38
Grazia Del Buono M 881
Graziano F 470
Greenberg ER 126, 714
Greenberg HS 157
Greenberg M 1219
Gregory WM 1293
Grenman R 659
Greulich KM 72
Gridelli C 38
Griffin MJ 1029
Griffith H 24 (Suppl 1)
Griffith K 1460
Grigat JP 697
Griggs J 832
Gronberg H 429
Grossi F 1023
Grottola A 881
Gruber A 621
Gruenewald S 297
Grunfeld EA 1172
Grunicke H 1405
Guastalla JP 18 (Suppl 2),
24 (Suppl 2), 31 (Suppl 2)
Gugger M 1372
Guglielmi A 1023
Guignat L 808
Guillou P 1443
Gullick WJ 1322
Guo Z 791
Gurney EM 19
Gutheil JC 157
Guy M 520
Habu S 1235
Hagiwara A 824
Hagiwara T 737 (L)
Hagiwara Y 1564
Haglund C 1363
Haie-Meder C 24 (Suppl 2)
Haisma HJ 550
Hajkova R 374
Hakama M 164
Halbert G 465
Hale JP 1029
Halford S 1314
Hall AJ 1207
Hallberg L 1193
Haller AM 1179
Haller U 33
Halter R 813
Hamajima N 1199
Hamam SM 321
Hamamoto S 1640
Hamilton SR 1182
Hammerlid E 149
Hammond DW 499
Hancock B 1146
Hancock BW 465, 499, 1293
Haniuda M 25
Hanks GW 587
Hanna M 587
Hansson J 329, 1505
Hansson L-E 965
Hara I 859
Hara S 859
Hardcastle JD 1443
Hardy, J 88 (Suppl 2)
Harlow BL 714
Harmon D 17 (Suppl 1)
Harris AL 558, 1322
Harrison R 8
Hartge P 126
Hartley JA 1671
Hartmann JT 313
Hasan C 1453
Hasegawa M 25
Hashimura M 209
Hatch EE 126
Hattori Y 1076
Haugen A 226
Hauschild A 1036
Hawkins NJ 232
Hawtin PG 413
Hearn S 1043
Heatherington AC 11 (Suppl 1)
Hebert JR 994
Hedley D 367
Heemann U 1265
Heiberg I 686
Heighway J 1372
Helgesen K 1219
Hellmann K 959
Helmerhorst TJM 796
Hemminki K 388, 969, 990,
1466, 1505
Hendry WF 340
Henriksson R 429
Henry-Amar M 55 (Suppl 2)
Hepburn PM 19
Herbst AL 126
Hermans J 64
Heros D 157
Herrero R 1219
Herrington CS 1058
Hesketh R 832
Hettmer S 1453
Hey K 1460
Hietanen P 897
Higashiyama S 1377
Higgins CD 406
Highley M 452
Hildesheim A 1219
Hill BT 290
Hill RP 1280
Hill SA 626
Hiraiwa N 1258
Hirakawa H 851
Hirose K 1199
Ho MS 709
Hobson R 11
Hochhauser D 1671
Hodgson SV 435
Hoffstetter S 31 (Suppl 2)
Hofler H 202
Hofmann J 1405
Hogendoorn PCW 535
Holder AL 565
Holford TR 1472
Holli K 164
Hollingshead M 1424
Holm G 621
Holmberg E 429
Holwell SE 290
Honda T 25
Hong Y-K 1252
Hoover RN 126
Hopwood P 8
Horn E 344
Horsman MR 1070
Houba PHJ 550
Hough RE 499
Houlihan PS 1182
Houlston RS 1314
Houvenaeghel G 24 (Suppl 2)
Howell SB 157
Hsieh MY 709
Hu X 791
Huang S 743
Hulthen L 1193
Hunter JAA 1146
Hurny C 1156
Hutchinson M 1219
Hutchison CJ 512
Huuhtanen RL 244
Huusko P 116
Hynes H 17 (Suppl 1)
Iacoangeli M 529
Ikonen T 1344
Iliadis A 604
Im S-A 1252
Imai Y 737 (L), 886
Inada M 886
Inazawa J 824
Inoue H 276
Inoue M 1199
Inui K 263
Inui Y 886
Iqbal M 1064
Irmin L 478
Iselius L 429
Ishihara S 1640
Islam I 218
Islam-Ali BS 1648
Isogai S 1681
Isola J 164
Itamochi H 1551
Ito N 886
Ito Y 1242, 1377
Itoh K 915
Iversen T 1463
Iwamoto Y 768
Iwatsuki K 920
Izawa M 1258
Jack A 1293
Jack AD 1207
Jackson JA 1544
Jackson LE 691
Jacob JH 61 (Suppl 2)
Jacot W 903
Jaeckle KA 157
Jaehde U 1453
Jagannathan NR 1016
Jagdev SP 1126
Jaiyesimi IA 24 (Suppl 1)
Jakic-Razumovic J 1227
Jansen SJT 1577
Janssens MY 1122
Jansson O 329, 1505
Jarup L 1482
Author index 1689
British Journal of Cancer (2001) 84(12), 1687-1694
(c) 2001 Cancer Research Campaign
Jarvis C 1064
Jayson GC 465
Jelinek F 374
Jeng JE 709
Jenkins V 48, 1011
Jenkinson CM 413
Jensen OM 1070
Jerkeman M 303
Jeskanen L 659
Jewell RC 42
Jin F 994
Jodrell DI 600
Joensuu H 164
Johnson JI 1424
Johnson KJ 1076
Johnson PWM 19
Johnston A 11
Jolly K 1308
Jones A 558
Joo HJ 186
Jordan R 489
Jordan S 340
Jouve JL 1477
Julka PK 1016
Jung YD 844
Justice G 17 (Suppl 1)
Kagami I 545
Kainu T 116
Kallioniemi OP 1344
Kallioniemi O-P 116
Kalso E 587
Kalyandrug S 1424
Kamai T 1242
Kamazawa S 1551
Kameyama T 915
Kamidono S 859
Kamp S 1330
Kanamori M 1610
Kanamori Y 1551
Kaneko F 920
Kaneko S 400
Kannagi R 1258
Kanuma T 545
Kanz L 313
Kappas AM 1602
Kappeler A 1372
Karakostas K 1602
Karameris A 1488
Karoski WJ 1412
Kaskel P 72
Kassem HSh 321
Kasumi F 736 (L)
Kataoka A 276
Katballe N 1084
Kato H 851, 1235
Kato I 975
Katsaros D 643
Kaufman R 126
Kaufmann R 1036
Kaufmann SH 1391
Kavanagh M-C 1280
Kawakami S 25
Kawano Y 263
Kawashima K 1640
Kawata S 886
Kaye SB 170
Kazumori H 1640
Keck A-V 344
Keller J 1070
Kenani A 1272
Kerbrat P 18 (Suppl 2)
Kerkela E 659
Kessler J 17 (Suppl 1)
Key T 728
Khan F 1512
Khoo K-S 1185
Khuder SA 1188
Kievit J 1577
Kigawa J 1551
Kikuchi S 920
Kim H 643
Kim HC 186
Kim HS 186
Kim J-S 1252
Kim KB 186
Kim MS 844
Kim MW 186
Kim MY 975
Kimura T 1610
Kinlen LJ 1002
Kinoshita Y 1640
Kisseljov F 791
Klein Kranenbarg E 64
Kliukiene J 397
Klocke R 813
Klokman WJ 700
Knekt P 122
Knezetic JA 926
Knop S 313
Knowles G 600
Kodaira N 1681
Koenig KL 975
Koga T 915
Kogut N 1591
Kohler J 11
Kohler R 78 (Suppl 2)
Kohn EC 1330
Koivisto PA 1344
Koivunen E 1363
Konerding MA 1354
Kontani K 1258
Koopman FJ 42
Kornacker M 381
Kosmaczewska A 1339
Kovarik J 374
Kraehn GM 72
Kreienbrock L 134
Kreuzer M 134
Kringlen E 1463
Kroesen BJ 1115
Kuba K 864
Kubo K 25
Kulka U 202
Kulke MH 748
Kumar M 1016
Kuroishi T 1199
Kuter D 17 (Suppl 1)
Kvarnung M 122
La Vecchia C 722
Labianca R 470, 1023
Lacava J 651
Ladner DP 33
Laffargue F 31 (Suppl 2)
Lafitte JJ 1150
LaFollette S 157
Lagergren J 120
Laghi L 196
Lagrange JL 49 (Suppl 2)
Lake RA 52
Lancashire RJ 141
Landriscina M 529
Lane EB 512
Lane SR 1043
Lang JC 237
Lang SH 1417
Langezaal SM 535
Lansac J 31 (Suppl 2)
Lansdown MRJ 1095
Lapidus L 1193
Larsen SS 686
Larsson J 926
Larsson P 329, 1505
Lartigau E 24 (Suppl 2)
Lashford L 11
Lassen P 65 (Suppl 2)
Lastella M 1535
Lau JYW 335
Laurberg S 1084
Laurila P 244
Le Chevalier T 903
Leach MO 691
Leblanc E 24 (Suppl 2)
Lee AV 493
Lee C 100
Lee KB 186
Lee SN 1252
Lee T-L 335
Leenders RGG 550
Legrand E 802
Lehmann M 49 (Suppl 2)
Lehnert G 982
Leidecker V 8
Leiter U 72
Lejeune C 1477
Lemaitre F 1150
Leminen A 1363
Lemoine NR 253, 1656
Leonard RCF 1437
Leone B 651
Leone R 1387
Leong LA 1591
Lerouge D 1179
Lesser G 157
Letizia C 636
Leung WK 335
Levin B 1182
Levitz M 975
Lewandowicz GM 680
Lewis CM 435
Lewis IJ 11
Lhomme C 18 (Suppl 2),
24 (Suppl 2)
Li F 25
Li L 80
Li M 263
Li X 969
Liaras J 360
Lim H-Y 186
Limoli CL 489
Lin X 1571
Lin ZY 709
Linch DC 1293
Linder S 329
Lindner H 1330
Linse R 1036
Lister TA 147 (L)
Little J 910
Little MA 1029
Liu F 1556
Liu R 1405
Liu T-J 1252
Liu W 844
Loda M 748
Logue JP 8
Lohi J 659
Longmate J 1591
Longo G 303
Lorenz M 1602
Lorenzato M 1616
Lorigan PC 499
Lorincz AT 1219
Lorite MJ 1135
Lotan R 1528
Louwman WJ 700
Lovas S 926
Lu P 1405
Ludvikova V 374
Ludwig H 344
Lukas P 179
Lukish JR 1182
Lund Nilsen TI 417
Lunec J 270
Luny D 512
Lupoli G 1586
Lykkesfeldt AE 686
Maartense E 1577
McCann J 423
Macchia M 1535
McDowell H 11
McGill A 452
McGown AT 465
Machin D 1185
Mackie PS 951
Mackie RM 1146
McKinney PA 141
McLaren BM 52
McLean C 600
McLean S 512
Maclennan KA 1293
McLeod HL 308
McQuay HJ 587
Maehara N 864
Maher EJ 1172
Maislinger S 179
Makela P 1047
Malcolmson AM 674
Malesci A 196
Malingre MM 42
Malmer B 429
Manenti F 881
Manfredi MG 1107
Mangeonjean C 1616
Mann JR 141
Manno M 881
Manzione L 38
Manzotti C 1387
Marchetti A 1535
Margison GP 321
Margolin KA 1591
Maria B 157
Maric R 1215
Marinov J 374
Marko D 283
Marshall J 1599
Martignetti A 1586
Martin PM 783
Martin P-M 936
1690 Author index
British Journal of Cancer (2001) 84(12), 1687-1694 (c) 2001 Cancer Research Campaign
Marttunen MB 897
Maruyama Y 25
Masaki T 1317
Mascaux C 1150
Mason W 157
Massobrio M 643
Massoudi N 381
Masters JRW 1686 (BR)
Mastrangelo R 460
Mastrangelo S 460
Masuda K 824
Masuda S 836
Masunaga H 1681
Masure M 1616
Mathoulin S 78 (Suppl 2)
Matikainen M 1344
Matsuda K 94, 1052
Matsuda S 768
Matsuda Y 886
Matsumoto K 864
Matsumoto Y 768
Matsumura Y 1235
Matsunobu T 768
Matsuo T 836
Matsuoka H 1317
Matsuura N 1377
Matsuzawa Y 886
Mattes RH 1330
Matthews CE 994
Maula S 244
Maxwell-Armstrong CA 1443
Mayer RJ 441 (L)
Mayne ST 1472
Mayumi T 1564
Mazurenko N 791
Mazzanti R 878
Meagher A 232
Medi F 878
Meert AP 1150
Meijer CJLM 796
Meijer L 283
Mellon JK 270
Melloni E 946
Meng XL 1544
Mercadante S 587
Mercatelli A 878
Mercer AJ 11 (Suppl 1)
Merrouche Y 65 (Suppl 2)
Messori A 303
Meta-Analysis Group in
Cancer 611
Metastron Users Group 297
Metcalfe JC 832
Meynadier J 587
Meza LA 24 (Suppl 1)
Mezzelani A 1087
Michael BD 674
Michaelis J 697
Middleton M 1286 (BR)
Middleton MR 1141
Milano G 579
Mir LM 1272
Mitsudomi T 1199
Mittal KR 975
Mittra I 1286 (BR)
Miura S 1258
Miyahara R 263
Miyake H 859
Miyoshi E 1377
Mizumoto K 864
Mizunuma H 545
M-Kahari V 659
Mohr P 1036
Mohsin SK 493
Mok SC 352
Mokhtar N 1182
Moll I 819
Moller H 435
Monden M 1377
Monges G 65 (Suppl 2),
74 (Suppl 2)
Monnier A 1179
Mononen N 1344
Monsaert C 1122
Monson JRT 1443
Monsonego J 360
Moore MJ 367
Moore PS 253
Moorghen M 520
Moots P 157
Morales J 1219
Morat L 504
Moreau L 903
Moretti A 636
Moretti R 878
Moretti S 604
Morgan J 80
Morgan Jr RJ 1591
Morgan WF 489
Mori K 1564
Mori M 276
Mori T 1235
Morilla R 1339
Morland B 11, 1029
Morley S 512
Morris TM 1664
Mosca F 1535
Moteleb E 512
Mottot C 360
Mousses S 1635
Muci D 1166
Mueller SC 1330
Mueller W 199
Muers MF 19
Muir G 1512
Muir KM 413
Mukherjee P 1107
Mulder NH 1348
Murai H 1258
Murakami A 851
Murdock M 24 (Suppl 1)
Murphy HS 1076
Murphy LO 926
Murphy MFG 1460
Murphy RF 926
Murray P 1227
Musha T 836
Musk AW 52
Mutanen P 990, 1466
Mutgi AB 1188
Muzzammil T 367
Nagao M 836
Nagase T 886
Nagasue N 1640
Nakagawa S 915, 1564
Nakajima S 1681
Nakamura T 864
Nakamura Y 824, 836
Nakanishi M 824
Nakatani F 768
Nam DK 186
Napp V 19
Narita T 1258
Nashan D 1036
Natale M 1166
Nawata S 851
Nazeyrollas P 1616
Neal DE 270
Negri E 722
Negrier S 81 (Suppl 2)
Nehme A 1571
Neri B 878
Neuzil J 87
Nevanlinna H 116, 704
New P 157
Newell DR 11, 106, 1289
Ng E-H 1185
Ng EKW 335
Ng Y-P 335
Nicolella G 1166
Niedner H 1571
Nielsen OS 1070
Niklasson A 1193
Nister M 791
Nobbenhuis MAE 796
Nocera M 808
Noda K 1377
Noda S 886
Noller K 126
Noma H 754
Nooij MA 1577
Nordsmark M 1070
Noss A 571
Nukaya I 94
Numa F 851
Nuzzo V 1586
Nyren O 120, 965
O'Byrne J 17 (Suppl 1)
O'Connell P 493
O'Day SJ 157
Odze RD 748
Oesterreich S 493
Ogawa M 1497
Ogawa N 443
Ohmori K 1610
Ohtsuka M 920
Oishi T 1551
Okada N 1564
Okamoto T 1235
Okayasu I 209
Okuyama T 1640
Oleinick NL 1099
Oliver RT 340
Ollivier JM 61 (Suppl 2)
On On Chan A 1182
Orbetegli O 196
Orita K 443
Orlandini S 253
Orlowski S 1272
Orntoft T 1084
Osborne CK 493
Oshima H 1242
Otake Y 263
Otvos F 926
Ouafik LH 783
Ouyang N 1265
Overgaard J 1070
Overpelt IME 1577
Owens PH 1472
Oyama N 920
Ozcelik H 1635
Paesmans M 1150
Pages S 482
Pairon JC 49 (Suppl 2)
Paju A 1363
Pallaska A 1397
Palmari J 783
Palmer J 126
Palmeri S 1166
Pang T 791
Pani G 529
Panza N 1166
Papworth R 776
Paradiso A 651
Paradiso L 308
Paraschou P 1602
Paraskeva C 520
Parkin D 170
Parkin DM 1207
Parkins CS 565
Parkinson EK 1630
Parnaud G 90
Parry A 1029
Parry J 1308
Paskins L 1512
Patel A 1512
Patricot LM 78 (Suppl 2)
Paul D 813
Paul EM 42
Paul J 170
Paysant J 802
Peach D 1172
Peake MD 19
Pearson ADJ 11, 1029
Pecherstorfer M 344
Pedley RB 1671
Pegelow K 1330
Peiffer G 90
Pein F 604
Pennati M 1387
Perabo FGE 1330
Perez K 714
Pergolizzi R 1528
Perie S 504
Permert J 926
Perrone F 38
Perucho M 743
Pessi MA 1023
Pession A 460
Peter RU 72
Peterse JL 539
Philip T 4 (Suppl 2),
55 (Suppl 2), 78 (Suppl 2)
Phuphanich S 157
Piantedosi F 1166
Piao Z 743
Piccinno R 643
Pierotti MA 1087
Pignatelli M 1656
Pigneux J 31 (Suppl 2)
Pilotti S 539, 1087
Pincheira R 1520
Pinedo HM 550
Pinkerton CR 11
Pinsolle J 37 (Suppl 2)
Pithavala Y 308
Pitsiladis M 11, 308
Author index 1691
British Journal of Cancer (2001) 84(12), 1687-1694
(c) 2001 Cancer Research Campaign
Platt FM 1107
Ponten J 791
Portwood N 329
Poulain P 587
Poulter KM 19
Povysil C 374
Prete A 460
Price E 1029
Price L 11
Prise KM 674
Prow D 17 (Suppl 1)
Puisieux A 193
Pujol J-L 903
Purdie D 352
Putaud I 1616
Pyrhonen S 897
Qi H 1556
Quantin X 903
Quereux C 1616
Quinlan RA 512
Quoix E 903
Radbruch L 587
Radford JA 465
Raju U 975
Ramirez AJ 1172
Ranes MK 1107
Ranson M 465
Rapakko K 116
Rarick M 24 (Suppl 1)
Raschko JW 1591
Ratashak A 1412
Ratcliffe D 1011
Rath GK 1016
Rath P 475
Raoul JL 74 (Suppl 2)
Ravaioli A 1023
Ray I 18 (Suppl 2)
Raymond E 631
Reardon D 1591
Recaldin E 1023
Recht L 157
Reed MWR 1384
Reed NS 170
Regnard JF 51
(Suppl 2)
Reif S 1453
Remiddi F 529
Remontet L 1477
Renault-Salis JL 37
(Suppl 2)
Resbeut M 24 (Suppl 2),
51 (Suppl 2)
Reymond MO 783
Ria F 529
Ricci S 636
Richards D 17 (Suppl 1)
Richards MA 1172
Ricker W 126
Rigacci L 303
Rigas J 17 (Suppl 1)
Rigaud G 253
Riordan HD 1544
Riordan NH 1544
Ripamonti C 587
Risse EKJ 796
Ristamaki R 244
Ritchie LD 910
Riva C 1087
Riviere A 1179
Rivoire M 65 (Suppl 2)
Robanus-Maandag EC 539
Robbins A 465
Roberts SA 776, 892
Robins RA 1443
Robinson BWS 52
Robinson D 435
Roca i Casas J 587
Rodier JF 31 (Suppl 2)
Rodriguez AC 1219
Rogers L 157
Rohatiner AZS 147 (L)
Romain S 783
Romano S 878
Roncalli M 196
Ronen SM 691
Rose C 783
Rosing H 42
Ross G 1043
Rosselli R 529
Rossi A 38
Rostagno P 579
Rostami-H A 1126
Rougier P 61 (Suppl 2), 65
(Suppl 2), 74 (Suppl 2)
Roukos DH 1602
Rouquet C 1677
Rouse A 1308
Roussel A 61 (Suppl 2)
Rowan A 1314
Roy HK 1412
Rozendaal L 796
Ruan Z-X 994
Rubinstein LV 1424
Rudenstam C-M 1156
Rufini V 460
Ruffie P 49 (Suppl 2),
51 (Suppl 2)
Ruggieri-Pignon S 65 (Suppl 2)
Ruiters MHJ 1348
Rumi MAK 1640
Rumpold G 179
Rush R 594
Russell C 157
Russo A 1166
Rustin G 1043
Ryan DP 441 (L)
Rybczynska M 1405
Ryberg D 226
Saarialho-Kere U 659
Saaristo R 164
Saarto T 1047
Sabatier L 504
Saegusa M 209
Saga T 1681
Saito M 736 (L)
Saito T 1564
Saito Y 1235
Sakahara H 1681
Sakakura C 824
Sakamoto A 1317
Sakata I 1681
Sakon M 1377
Salo T 1363
Salvetti A 636
Sambrook RJ 1447
Sandstedt B 1505
Sanghvi V 739
Saranath D 739
Sargent JM 680, 959
Sarradat A 61 (Suppl 2)
Sasaki R 836
Sasatomi T 915
Sastre-Garaud X 24 (Suppl 2)
Sato H 1640
Sato S 1551
Satoh M 920
Saul J 48, 1011
Saurel J 360
Sausville EA 1424
Saven A 17 (Suppl 1),
24 (Suppl 1)
Sawe J 587
Scarpa A 253
Scerrati M 529
Schally AV 926
Schatzle S 283
Scheeren JW 550
Schellens JHM 42
Schepartz S 1424
Schiffman M 1219
Schleutker J 1344
Schlumberger M 808
Schmidt D 1330
Schmidt T 1076
Schmidt TL 1544
Schmitz U 199
Scholefield JH 1443
Schriber J 1591
Schroder CP 1348
Schuller DE 237
Schuller J 1043
Schuller J 11 (Suppl 1)
Schulz TF 122
Schumacher U 819
Schuz J 697
Schwartz JL 489
Schwartzberg L 17 (Suppl 1)
Scorilas A 643, 760, 1488
Scott D 776, 892
Scott LJ 1417
Scottish Gynaecological Cancer
Trials Group 170
Scottish Melanoma Group 1146
Sculier JP 1150
Sebastian G 1036
Sebban C 55 (Suppl 2)
Seddighzadeh M 329
Seenu V 1016
Seibel MJ 344
Seifeldin IA 1182
Seigneurin JM 783
Seiter S 1036
Seitz JF 61 (Suppl 2)
Selby P 1293
Sellakumar G 1571
Senoo M 1235
Seong C-M 1252
Sersa G 565
Sette A 94
Sevenet N 199
Seyfried TN 1107
Shamash J 340
Shanks JH 1417
Shapiro W 157
Sharma SK 1671
Sharom FJ 1405
Sharp L 910
Sheedy B 308
Sherman ME 1219
Sheu JC 743
Shibahara T 754
Shibata SI 1591
Shichijo S 915
Shimada M 1551
Shimada S 1497
Shimomura K 824
Shin BA 844
Shiomori K 1497
Shipman CM 1126
Shoker BS 1064
Shore RE 975
Shu X-O 994
Sibson DR 1064
Signorello LB 965
Silcocks P 728, 1215
Siliani L 878
Silvy M 936
Simon D 743
Simone G 651
Simova J 374
Singh Jadeja J 17 (Suppl 1)
Sivagnanasundaram S 218
Skaug V 226
Skliris GP 1095
Slevin N 776
Slevin NJ 8
Sloane JP 1064
Smahel M 374
Smith GA 832
Smith K 1322
Smith P 1293
Smith RE Jr 24 (Suppl 1)
Smyrk TC 1412
Smyth JF 1146
Snaddon J 1630
Snijders PJF 1058
Sobotkova E 374
Sobrero A 1023
Soletormos G 1301
Soliman AS 1182
Sollner W 179
Somlo G 1591
Sone S 25
Song E 1265
Sorahan T 141
Soria C 802
Soria JC 504
Soria J-C 631
Sorsa T 1363
Soukop M 465
Soussi T 57
Sozzi G 1087
Speirs V 1095
Spendlove I 80
Spitaler M 1405
Sprinzl G 179
Spry N 297
Spyratos F 783
Srivastava A 1016
Stanson J 1624
Staratschek-Jox 381
Steel CM 594
Steels E 1150
Steineck G 329, 1505
Steiner G 1330
Steiner RA 33
Steinfels E 1405
1692 Author index
British Journal of Cancer (2001) 84(12), 1687-1694 (c) 2001 Cancer Research Campaign
Steixner E 179
Stel AJ 1115
Stenman U-H 1363
Stevens A 1308
Stevenson S 52
Stewart M 600
Stiggelbout AM 1577
Stiller CA 141, 406
Stines J 55 (Suppl 2)
Stolte H 313
Stolz W 1036
Stoppa-Lyonnet D 482
Storme G 6 (Suppl 2)
Storme GA 1122
Suefuji Y 915
Sugimachi K 276
Sugiura T 1199
Sugiyama M 1317
Sulkes J 478
Sullivan M 1156
Suminami Y 851
Sun XS 1179
Sung JJY 335
Sweetenham J 1291
Swerdlow AJ 406
Swinnen L 157
Synold T 1591
Syrjakoski K 116, 1344
Szekely L 122
Tache S 90
Tada A 1564
Tada T 736 (L)
Taft C 149
Taguchi O 1258
Tajima K 1199
Takagi K 1242
Takahashi K 736 (L)
Takahashi M 1551
Takama F 545
Takashima S 25
Takeda H 1681
Takeda T 1377
Takehara Y 1681
Takesako K 94
Takeuchi T 1258
Takezaki T 1199
Talbot I 218
Talieri M 1488
Tamborini E 1087
Tammela TLJ 1344
Tamminen A 704
Tamura S 886
Tamura Y 1564
Tanaka F 263
Tanaka H 94, 836, 1052
Tanaka K 768
Tanaka M 864
Tanaka N 443
Tandle AT 739
Tanimura H 94, 1052
Tannapfel A 982
Tannock IF 100
Tanzawa H 754
Tarquini R 878
Tashima S 1497
Taylor CG 680, 959
Taylor DD 1624
Taylor GA 11
Tchekmedyian NS 24 (Suppl 1)
Tedeschi R 122
Ten Bokkel Huinink WW 42
Teppo L 122
Terakawa N 1551
Ternier F 24 (Suppl 2)
Terry P 120
Tervahartiala T 1363
Testa NG 1417
Testore P 1023
Tetef ML 1591
Thakore KS 748
Thatcher N 1141
Theobald S 8 (Suppl 2)
Theodor L 475
Thibout D 802
Thies A 819
Thiesse P 78 (Suppl 2)
Thomas G 748
Thomas GA 832
Thomas L 18 (Suppl 2),
31 (Suppl 2)
Thomas RK 381
Thornalley PJ 670
Tiitinen A 897
Tilgen W 1036
Tisdale MJ 1135, 1648
Titus-Ernstoff L 126, 714
To K-F 335
Toledano MB 1482
Tominaga O 57
Tomlinson I 232
Tomlinson IPM 1314
Toniolo P 975
Tornesello A 460
Torres-Duarte A 1599
Tosi M 482
Tounekti O 1272
Tourne ME 1391
Tournemaine N 18 (Suppl 2)
Tozer GM 565
Tranga T 1488
Tretli S 1463
Trippoli S 303
Troncone L 460
Tsai JF 709
Tsuchiya I 1235
Tsujimoto M 1377
Tsujino M 1564
Tsunoda T 94, 1052
Tsuruta J 1497
Turci D 1023
Turner SL 297
Tut VM 270
Tuxen MK 1301
Twycross RG 587
Tynes T 397
Udart M 72
Ugurel S 1036
Ulrich J 1036
Umano Y 94, 1052
Underwood M 1512
Unsal K 57
Utikal J 72
Utsumi H 836
Uzawa K 754
Vahakangas K 116
Vahteristo P 116, 704
Vaiani M 303
Vaittinen P 388
Valimaki M 1047
Valle JW 875
Vallejo C 651
Vallot F 1150
van de Velde CJH 64
van de Vijver MJ 539
van den Bent MJ 157
Van den Berge DL 1122
van den Brule AJC 796
van der Graaf WTA 1348
van der Meulen-Muileman IH
550
van der Zee AGJ 1348
van Duin M 1058
van Krieken JHJM 64
van Lohuizen M 1372
van Slooten H 1577
Van Tellingen O 604
Varadarajan P 1571
Varani J 1076
Varghese C 1215
Varley JM 321
Vasey PA 170
Vassal G 604
Vasse M 802
Vatten LJ 417
Vaughan Hudson G 1293
Veal GJ 1029
Vehmanen L 1047
Veltri E 38
Venables RS 512
Venditti JM 1424
Ventafridda V 587
Verheijen RHM 796
Verovski VN 1122
Vian L 1677
Villa E 881
Vilmer C 81 (Suppl 2)
Vincent P 31 (Suppl 2)
Vincent TJ 1460
Vitale G 1586
von Deimling A 199
von Ruecker A 1330
von Vangerow A 313
Vonka V 374
Vonlanthen S 1372
Voog E 18 (Suppl 2)
Voorhorst FJ 796
Wacholder S 1219
Wada H 263
Wahlstrom T 897, 1363
Wainer IW 1599
Wakefield J 1482
Walch A 202
Walker M 1512
Wall L 600
Wallgren A 1193
Walt H 33
Walters JRF 218
Wang D 545
Wang H 748
Wang LY 709
Wang M 1265
Wang Q 193
Ward RL 232
Warnes SL 571
Warren C 892
Wasan H 1314
Watanabe T 1182
Waters J 1
Watson SA 1664
Weber C 87
Weber M 199
Weber T 87
Webley SD 1671
Weihrauch M 982
Welbank H 1029
Wellmann J 134
Werner M 202
West CM 892
Whiteman D 1460
Whiteside TL 1624
Whitley E 728
Whittle H 1207
Wichmann HE 134
Wijkstrom H 329, 1505
Wiklund TA 244
Wikman FP 1084
Williams AC 520
Williams ED 832
Williamson CJ 680, 959
Winqvist R 116
Winter H 1215
Wirger A 1330
Wisman GBA 1348
Withoff S 1115
Wittekind C 982
Woitge HW 344
Wojno KJ 1076
Wolf J 381
Wolk A 120
Wong E 157
Wong H 493
Woodford-Richens KL 1314
Wrbitzky R 982
Wright JG 452
Wunder JS 1635
Wyld L 1384
Xu D 621
Xu K 670
Xu Z-G 920
Yakushiji T 754
Yamada T 263
Yamagishi H 824
Yamamoto A 1564
Yamamoto N 754
Yamanda T 25
Yamasaki E 886
Yamashita 276
Yamaue H 94, 1052
Yanagihara K 263
Yang Z-G 25
Yardley C 959
Yasuda T 1610
Yasuda Y 836
Yasuoka R 824
Yatabe Y 1199
Yen Y 1591
Yeremin L 475
Yi JW 186
Yi Q 621
Ylikorkala O 897
Yoshida K-I 1242
Yoshida T 209
Yoshimoto M 736 (L)
Yoshimura T 400
Author index 1693
British Journal of Cancer (2001) 84(12), 1687-1694
(c) 2001 Cancer Research Campaign
Young T 1172
Yousef GM 643
Yudoh K 1610
Yule SM 1029
Yung WKA 1252
Zaffaroni N 1387
Zaharevitz D 1424
Zahm SH 1472
Zak R 374
Zalcman G 57
Zamboni G 253
Zander T 381
Zankl H 283
Zeleniuch-Jacquotte A 975
Zenita K 1258
Zerat L 360
Zhang B 1472
Zhang J-T 1520
Zhang Q 493, 1571
Zhang Y 1472
Zheng C 621
Zheng T 1472
Zheng W 994
Zhou H 951
Ziegler R 344
Zienolddiny S 226
Zitzelsberger H 202
1694 Author index
British Journal of Cancer (2001) 84(12), 1687-1694 (c) 2001 Cancer Research Campaign

				

